# Treatment With Nimodipine or FK506 After Facial Nerve Repair Neither Improves Accuracy of Reinnervation Nor Recovery of Mimetic Function in Rats

**DOI:** 10.3389/fnins.2022.895076

**Published:** 2022-05-13

**Authors:** Mohammed Barham, Michael Streppel, Orlando Guntinas-Lichius, Nicole Fulgham-Scott, Johannes Vogt, Wolfram F. Neiss

**Affiliations:** ^1^Department II of Anatomy, Faculty of Medicine, University of Cologne and University Hospital of Cologne, Cologne, Germany; ^2^Department of Ear, Nose and Throat-Department (ENT), PAN-Clinic at Neumarkt, Cologne, Germany; ^3^Department of Otorhinolaryngology, Jena University Hospital, Jena, Germany; ^4^Department I of Anatomy, Faculty of Medicine, University of Cologne and University Hospital of Cologne, Cologne, Germany

**Keywords:** Fast-Blue, FK506, Fluoro-Gold, motion analysis, motoneuron, misdirected reinnervation, nimodipine

## Abstract

**Purpose:**

Nimodipine and FK506 (Tacrolimus) are drugs that have been reported to accelerate peripheral nerve regeneration. We therefore tested these substances aiming to improve the final functional outcome of motoric reinnervation after facial nerve injury.

**Methods:**

In 18 female rats, the transected facial nerve was repaired by an artificial nerve conduit. The rats were then treated with either placebo, nimodipine, or FK506, for 56 days. Facial motoneurons were pre-operatively double-labeled by Fluoro-Gold and again 56 days post-operation by Fast-Blue to measure the cytological accuracy of reinnervation. The whisking motion of the vibrissae was analyzed to assess the quality of functional recovery.

**Results:**

On the non-operated side, 93–97% of those facial nerve motoneurons innervating the vibrissae were double-labeled. On the operated side, double-labeling only amounted to 38% (placebo), 40% (nimodipine), and 39% (FK506), indicating severe misdirection of reinnervation. Regardless of post-operative drug or placebo therapy, the whisking frequency reached 83–100% of the normal value (6.0 Hz), but whisking amplitude was reduced to 33–48% while whisking velocity reached 39–66% of the normal values. Compared to placebo, statistically neither nimodipine nor FK506 improved accuracy of reinnervation and function recovery.

**Conclusion:**

Despite previous, positive data on the speed and quantity of axonal regeneration, nimodipine and FK506 do not improve the final functional outcome of motoric reinnervation in rats.

## Introduction

After successful reconstruction of a damaged peripheral nerve by microsurgery (reviews Mackinnon, 1988; Millesi, 1992; Lundborg, 2004), two major problems arise. The first one concerns the slow axonal elongation rate of around 1 mm/day in human subjects. An acceleration of reinnervation has not yet been accomplished in clinical practice. Some candidate drugs that speed up regeneration after nerve repair in animal experiments are the 1,4-dihydropyridine L-type-Ca^2+^-channel-antagonist nimodipine (Angelov et al., 1996, 1997; Mattsson et al., 1999, 2001; Hydman et al., 2007; Nishimoto et al., 2012; Bork et al., 2015; Jia et al., 2015; Sin et al., 2018; Lin et al., 2019; Odorico et al., 2021), and the immunosuppressant FK506 (Tacrolimus, Prograf^®^) (Gold et al., 1995; Doolabh and Mackinnon, 1999; Fansa et al., 1999; Wang and Gold, 1999; Jost et al., 2000; Lee et al., 2000; Navarro et al., 2001; Chunasuwankul et al., 2002; Sulaiman et al., 2002; Udina et al., 2003, 2004; Brenner et al., 2004; Konofaos and Terzis, 2013; Labroo et al., 2016; Jo et al., 2019; Tajdaran et al., 2019).

We tested nimodipine and FK506 concerning the problem of misdirection of innervation (aberrant reinnervation). Due to random sprouting of regenerating motor, sensory, and vegetative axons in adult mammals, full functional recovery is still not obtainable (Anonsen et al., 1986; de Medinaceli, 1988; Millesi, 1993, 2006; Vaughan and Richardson, 1993; Amara et al., 2000). This phenomenon occurs due to the motor axons missprout from the proximal nerve stump into inappropriate distal pathways (Langley and Hashimoto, 1917; Esslen, 1960). The misdirected reinnervation of muscles (Thomander, 1984; Aldskogius and Thomander, 1986; Sumner, 1990) may lead to autoparalytic syndrome (Montserrat and Benito, 1988), antagonistic inhibition (Angelov et al., 1999; Dohm et al., 2000; Valero-Cabré et al., 2001, 2004; Valero-Cabré and Navarro, 2002; Hamilton et al., 2011), and synkinesis (Crumley, 1979; Montserrat and Benito, 1988; Yian et al., 2001; Harris et al., 2019).

The real challenge in peripheral nerve repair is to improve accuracy of reinnervation and thus to enhance functional recovery (review Abrams and Widenfalk, 2005). For this field of research, the facial nerve in rats is an optimal model since it is easily accessible and almost entirely consists of motor axons. The different branches of motor innervation can be visually identified by retrograde labeling (Thomander, 1984; Angelov et al., 1999; Dohm et al., 2000; Moran and Graeber, 2004). The mimetic function can readily be assessed by motion analysis of the sensory vibrissae (Guntinas-Lichius et al., 2002; Tomov et al., 2002; Angelov et al., 2007; Sofroniew and Svoboda, 2015).

According to previous studies, both nimodipine and FK506 have been shown to accelerate and to improve axon regeneration (Konofaos and Terzis, 2013; Bork et al., 2015; Huang et al., 2017; Jo et al., 2019). Whether this acceleration merely speeds up the misdirection of reinnervation or indeed improves accuracy has not yet been investigated. Using motion analysis of the whiskers and pre- and post-operative fluorescent double-labeling of motoneurons to investigate the accuracy of reinnervation (Brushart, 1990; Molander and Aldskogius, 1992; Madison et al., 1996; Popratiloff et al., 2001; Katada et al., 2006; Grosheva et al., 2008; Ozsoy et al., 2014; Placheta et al., 2015; Sofroniew and Svoboda, 2015), we tested whether treatment with either nimodipine or FK506 following facial nerve surgery in rats may contribute to a decrease in misdirection of reinnervation and to better functional recovery.

## Materials and Methods

### Experimental Design

Eighteen young adult rats received a first, preoperative intramuscular microinjection of Fluoro-Gold at precisely the same spot into the whisker pad (details see section “First, Preoperative Retrograde Labeling”) on both sides of the face, which specifically labels the motoneurons of the left and right lateral facial subnucleus (see Figure 2 in Angelov et al., 1993, HRP-labeling). 10 days later—transport time for the retrograde tracer—all rats were identically operated on the right side only. The main trunk of the facial nerve was transected distal to the posterior auricular nerve branch and repaired by artificial conduit (details see section “Nerve Transection and Repair by Implantation of Artificial Nerve Conduit”). Thereby all facial motoneurons, except those of the medial facial subnucleus which innervate the posterior and superior auricular muscles (Papez, 1927; Martin and Lodge, 1977), were axotomised, hence the complete musculature of all vibrissae was paralyzed. Immediately afterward, the animals were divided into 3 groups of 6 rats each for placebo treatment (PT), nimodipine treatment (NT), and FK506 treatment (FKT). Each treatment (details see section “Pharmacotherapy or Placebo Treatment Was Started Immediately After Nerve Surgery”), lasted for 56 days post-operation, as we have previously shown (see Figure 2 “Time course of reinnervation” in Streppel et al., 1998) that 56 days after facial-facial anastomosis, the number of regenerated motoneurons innervating the whisker pad equals that of normal anatomy, albeit with severe misdirection of reinnervation (Figure 1 in Streppel et al., 1998). At the end of regeneration time, we measured the regain of whisking of the vibrissae by motion analysis (details see section “Biometric Analysis of Whisking”). Immediately after the video recordings, all rats received a second, post-operative intramuscular microinjection of Fast-Blue as a differently colored fluorescent tracer again on both sides of the face at exactly the same spot of the whisker pad as the first, preoperative tracer had been applied (details see section “Second, Post-operative Retrograde Labeling”). 10 days later—transport time for the second retrograde tracer—i.e., 66 days post-surgery all rats were identically fixed by transcardial perfusion (details see section “Fixation, Tissue Processing and Serial Sectioning”), the brainstem removed and prepared as serial sections for neuron counting.

**FIGURE 1:**
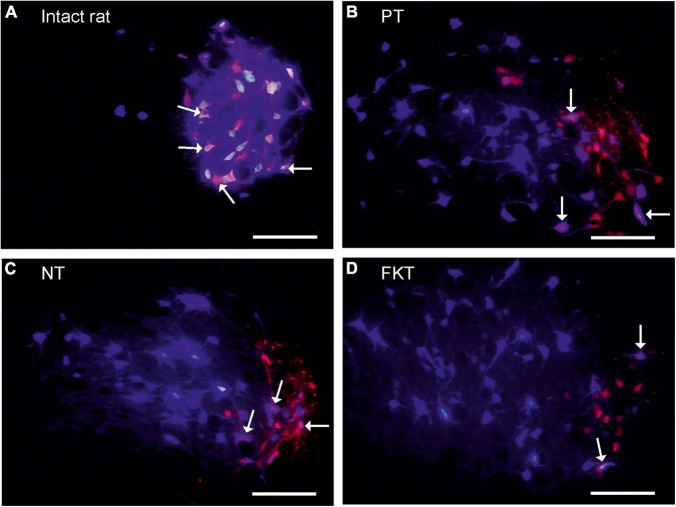
Labeling of facial motoneurons. The original motoneurons of the lateral facial subnucleus that in normal anatomy innervate the vibrissae muscles, were retrogradely (pre)-labeled *red* by injection of Fluoro-Gold (FG) into the whisker pad. The regenerated motoneurons that innervate the whisker muscles following reinnervation were retrogradely post-labeled *blue* by injection of Fast Blue (FB) at the same site 56 days after facial nerve repair, 10 days prior to fixation All images addition of FG-selective filter + FB-selective filter from same field of view, primary magnification objective 10×, Scale bar 100 μm. **(A)** In intact rat, 94% of the motoneurons prelabeled with FG are double-labeled also with FB (*arrows*). This is the normal myotopic organization in the lateral facial subnucleus. **(B)** Implantation of an artificial nerve conduit (AC) into the facial nerve trunk, post-operative treatment with placebo (PT). On the operated side all preoperatively labeled facial motoneurons (*red*) are exclusively localized in the lateral facial subnucleus which is the normal myotopic distribution, but the post-operatively labeled motoneurons (*blue*), are scattered throughout the entire facial nucleus (except for the medial subnucleus) proving a complete loss of myotopic organization and severe misdirection of reinnervation. Only 38% motoneurons are double-labeled (*pink*, *arrows*). **(C,D)** Implantation of an artificial nerve conduit followed by systemic drug therapy with nimodipine (NT, **C)** or FK506 (FKT, **D)** for 56 days. About 40% of the neurons are double-labeled (*pink*, *arrows*). There is no difference in number and distribution pattern of labeled motoneurons to that of placebo-treated rats **(B)**.

### Experimental Animals

Eighteen young adult, female Wistar rats, weighing 175–200 g, strain HsdCpb: WU (Harlan Winkelmann, D-33176 Borchen, Germany) were fed standard laboratory chow (Ssniff, D-59494 Soest, Germany) and tap water *ad libitum*. They were kept in type IIIH cage systems [L 425 mm × W 265 mm × H 180 mm with a floor area of 800 cm^2^; two animals per cage (bioscape)] in the animal house of the Department of Anatomy of the Cologne University Hospital. The animal house has an air exchange rate of at least 8× per hour; the temperature range is between 22 and 24°C and the relative humidity 45–65% rH. The artificial light-dark cycle is 12-h on-off. All experimental procedures were performed according to the guidelines of the European Union Council (86/609/EU), and the Local Animal Protection Committee (Bezirksregierung Köln, Az. 50.203.2-K35, 34/2001) approved all experimental protocols.

### First, Preoperative Retrograde Labeling

In all animals labeling was performed by intramuscular injection of Fluoro-Gold. Under inhalation anesthesia (isoflurane 2%; Baxter GmbH Medication Delivery, 85716 Unterschleißheim, Germany), 100 μl of 1% Fluoro-Gold (FG; 1 mg FG in 100 μl sterilized distilled water containing 2% dimethyl sulfoxide; Fluorochrome Inc., Denver, Colorado, United States) was bilaterally injected into the whisker pad muscles. The injection was located at the mid-point between the two dorsal vibrissal rows A and B (Arvidsson, 1982; Angelov et al., 1993) and constituted the (first) preoperative fluorescence tracer. The FG retrogradely prelabeled the perikarya of all those motoneurons in red (Figures 1A–D), which extend axons that run through the buccal branch of the facial nerve and innervate the buccolabial muscles of the whisker pad (Angelov et al., 1999).

### Nerve Transection and Repair by Implantation of Artificial Nerve Conduit

The animals of all three groups (PT, NT, FKT) were identically operated with facial nerve transection and repair by artificial conduit. The intraperitoneal injection of anesthesia consisted of 0.05 ml Ketanest/Rompun per 10 g body weight [100 mg Ketanest (WDT, D-30827 Garbsen, Germany) plus 10 mg Rompun (Bayer AG, 51368 Leverkusen, Germany) per kg body weight; i.e., 1.0 ml Ketanest 100 mg/ml plus 0.5 ml Rompun 20 mg/ml mixed with 3.5 ml NaCl 0.9%]. Ten days after the preoperative labeling, the main trunk of the right facial nerve was transected. The transection occurred close to the stylomastoid foramen, while still maintaining distance from the posterior auricular nerve branch. The continuity of the nerve was immediately reconstructed by insertion of an artificial conduit (Forssman, 1898; Dahlin et al., 1988; Brushart et al., 1995; Valero-Cabré et al., 2001, 2004; Ozsoy et al., 2014), in order to compare our findings with other data on facial reinnervation after conduit implantation (Dohm et al., 2000; Guntinas-Lichius et al., 2001; Streppel et al., 2002; Ozsoy et al., 2014). Proximal and distal nerve stumps were inserted into the open ends of a small silicone tubes (inner diameter 1.47 mm, outer diameter 1.96 mm; Aromando Medizintechnik, Cat. No. 602-235, D-40213 Düsseldorf) with an interstump distance of 5 mm and the resulting 8.5 mmł regeneration chamber [5 mm × (0.735^2^×π) mm^2^], which was filled with collagen type I (100 μg/ml rat tail collagen, Serva No. 47254; Dohm et al., 2000). The nerve stumps were fixed to the conduit at both sides of the conduit with two stitches of resorbable suture material 10–0. The skin wound was closed with resorbable suture material 3–0.

### Pharmacotherapy or Placebo Treatment Was Started Immediately After Nerve Surgery

#### Placebo Treatment

For 56 days following the surgery, two rats per cage were fed Ssniff food pellets (standard formula rat/mouse; D-59454 Soest, Germany), i.e., no post-operative drug application.

#### Nimodipine Treatment

**FIGURE 2:**
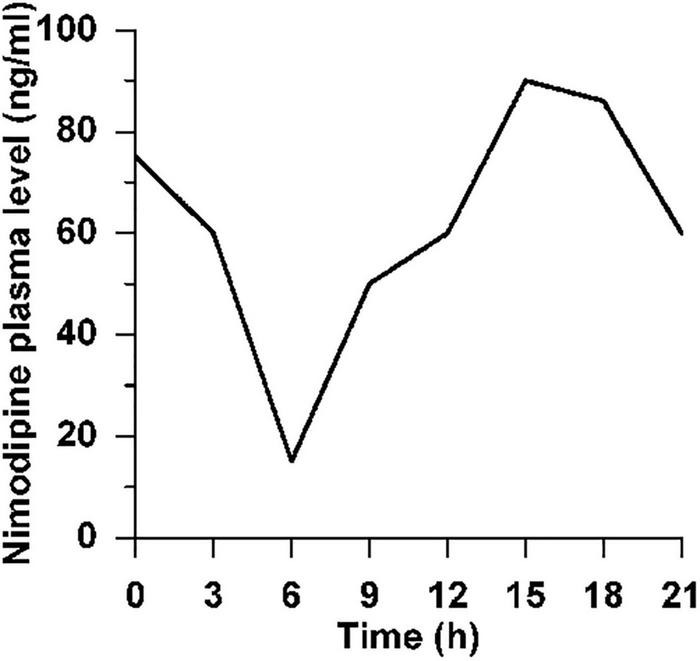
Circadian plasma level of nimodipine. Circadian plasma level of nimodipine after feeding of normal female Wistar rats *ad libitum* with 1,000 ppm nimodipine for 2 weeks. Animals were kept under artificial light at 0600–1800 h, in the dark 1800–0600 h. *N* = 4 rats per time point, hence no SD is indicated.

For 56 days, two rats per cage received Ssniff pellets containing 1,000 ppm nimodipine (donated by Bayer AG), supplied daily as fresh food because of the light sensitivity of nimodipine. The average daily food intake was 52 g/kg body weight/cage, i.e., 52 mg nimodipine/kg bw/day or about 10 mg nimdipine for a 200 g rat per day. In each of the 3 cages, the parallel weight gain of both rats during the 56 days treatment period did not indicate that one of the rats per cage had taken up more medicated food pellets than the other. This treatment led to a circadian plasma level of nimodipine ranging from 15 ng/ml at 0600 h to 90 ng/ml at 1500 h (light: midnight—noon, darkness: noon—midnight; Figure 2, unpublished data of W.F. Neiss in cooperation with Bayer AG).

As sham treatment in parallel to the FKT group, both PT and NT animals received 28 subcutaneous injections of the same volume of 0.9% saline during 56 days.

#### FK506 Treatment

Six rats were subcutaneously injected in the posterior cervical region with FK506 (Tacrolimus, Prograf^®^; donated by Fujisawa Co., Japan) with a mean dose of 0.5 mg FK506 per kg body weight per day. This dosage has been shown to function ideally in rats, acting as an accelerator of the peripheral nerve axon outgrowth, while simultaneously not inducing an immunosuppressant response (Fansa et al., 1999; Chunasuwankul et al., 2002; Udina et al., 2003; Yang et al., 2003). Each ampule contained 10 mg FK506 in 1 ml ethanol 95% plus vehicle (current packaging 5 mg/ml). This was diluted 1:10 with 9 ml 0.9% NaCl. Approximately 250 μl of this dilution was subcutaneously injected as a single dose every 48 h (cf. Jensen et al., 2005) at exactly 11:00 h (1 mg FK506/kg bw/48 h). Treatment was started on the day of conduit implantation and continued for the next 56 days (28 injections). Precise drug doses were adjusted weekly according to body weight changes.

### Biometric Analysis of Whisking

Motion analysis of the vibrissae (Tomov et al., 2002; Franchi et al., 2006; Ozsoy et al., 2014) was identically performed in all 18 operated rats; 56 days after artificial nerve conduit implantation, the movements of two large vibrissae of the C row on each side of the face were recorded. Under inhalation anesthesia (isoflurane 2%), all other vibrissae were clipped. Using a digital video camera (Panasonic NV DX-110 EG with AY-DVM 60 EK mini-cassettes), rats were videotaped for 3–5 min during active exploration. Selection criteria for the best 1.5 s film sequence were a stable position of the rat’s head combined with active whisking. The tip of the rat’s nose and the inner angles of both eyes were defined as reference points. Each vibrissa is represented in the spatial model by two points, its base and a point on the shaft 0.5 cm away from base. Employing this model, the following parameters were evaluated: (1) Protraction measured by the rostrally opened angle (in degrees) between the midsagittal plane and the hair shaft. Low angle values represented the maximal protractions. (2) The whisking frequency as cycles of protraction and retraction/s. (3) The amplitude (the difference between the maximal retraction and maximal protraction in degrees). (4) The maximal angular velocity during protraction in degrees/s, and (5) the maximal angular acceleration during protraction in degrees/s.

### Second, Post-operative Retrograde Labeling

After recording of the spontaneous vibrissae movement, all rats were subjected to 2% isoflurane anesthesia and were bilaterally injected with 100 μl of 1% Fast-Blue [EMS-Chemie, D-64823 Groß-Umstadt, 1 mg FB in 100 μl distilled water containing 2% DMSO (Dimethylsulfoxid)] into the whisker pad at the same site as the preoperative tracer. In order to compare the number of preoperatively labeled, post-operatively labeled and of preoperatively and post-operatively double-labeled motoneurons, each animal was cautiously injected to ensure identical conditions. The post-operative survival time of 56 days, in addition to 10 days for retrograde labeling, was identical for all groups.

### Fixation, Tissue Processing, and Serial Sectioning

66 days after facial surgery, all rats were fixed by transcardial perfusion. Under deep Ketanest/Rompun anesthesia, the vascular system was rinsed for 60 s with 0.1 M phosphate buffer (PBS) and fixed for 20 min with approximately 500 ml 4% paraformaldehyde in 0.1 M PBS. The indicator for acceptable fixation was palpable hardening of the perfused animal’s neck muscles and liver within a few minutes. The operation site was dissected free: In all animals the facial nerve stumps were still in place and a newly formed, regenerated nerve traversed the conduit. The brain and about 10 mm of spinal cord were carefully removed and post-fixed by immersion in the perfusion fixative at 4°C overnight. The brainstem was then removed and sectioned with a vibratome (Leica VT 1000-S, D- 35578 Wetzlar, Germany) into a complete series of 35–38 vibratome cross-sections (50 μm thick). The facial nucleus is typically about 29–33 vibratome sections long (mean 30.9 ± 1.4), with each section being 50 μm thick (Neiss et al., 1992; Guntinas-Lichius et al., 1994; Guntinas-Lichius and Neiss, 1996). The sections were collected and submerged in 0.1 M phosphate buffered saline, washed in distilled water for 5 min, and mounted on chrome-gelatin-coated glass slides, which were then air-dried and stored in the dark at 4°C until microscopic examination.

### Fluorescence Microscopy and Neuron Counting

Vibratome sections were observed through a Bandpass-filter set for Fast-Blue (AHF Analysentechnik, D-72074 Tübingen, Germany, no. F31-000; excitation D 436/10; beamsplitter 450 DCLP; barrier filter D 470/40) that allows recognition of only FB-labeled motoneurons (*blue* in Figures 1A–D). Observations through a HQ-Schmalband-filter set for Fluoro-Gold (AHF Analysentechnik, no. F36-050; excitation D 369/49; beamsplitter 400 DCLP; barrier filter HQ 635/30) visualized all motoneurons containing FG (*red* in Figures 1A–D). The fluorescence cross-talk between the tracers was restricted; no FG-labeled neurons were visible through the FB-selective filter set, however, very few intensely FB-labeled neurons could be seen through the FG-selective filter set. Two TIFF images were recorded for each field of view. The full size of the brainstem profile that contained labeled facial motoneurons was covered by tiling the image frames.

Images for neuron counting were recorded with a Zeiss Axioskop (Oberkochen, Germany), objective Plan-Neofluar 10 × and a CCD camera (DEI-470, Optronics Engineering, Goleta, CA, United States) within the first 24 h after cutting. Using the image analysis software Optimas 6.5 (Optimas Corporation, Bothell, WA, United States), the numbers of motoneurons labeled with FB or FG were counted, then the images were superimposed and the numbers of double-labeled neurons assessed (Rende et al., 1991; Popratiloff et al., 2001; Skouras et al., 2002; Tomov et al., 2002; Ozsoy et al., 2014). Employing the physical fractionator (Gundersen, 1986), all retrogradely labeled motoneurons with a visible cell nucleus were counted in every third section throughout the brainstem on both the labeled unoperated and operated side (Neiss et al., 1992; Valero-Cabré et al., 2004). The total numbers of motoneurons single or double-labeled with FG/FB were also calculated. Double labeling with the preoperative and post-operative tracer indicates axonal projection to the original target muscle. Single labeling indicates that during regeneration the axon and its branches have not re-reached the original target muscle, and thus have been misdirected.

The facial motoneurons were counted at 200× final magnification on the computer screen. To overcome inter- and intra-observer bias, neuron counting was exclusively performed in coded sections. The code for neuron counting was solved only after all raw data had been collected.

### Statistical Evaluation

In the present study, all values are expressed as the mean ± SD or the percentage of the total number of labeled motoneurons. The effects of nerve surgery and drug treatment on the electrophysiological (biometric analysis of whisking), and morphological (retrograde labeling of facial motoneurons) parameters of all rats were analyzed using one-way analysis of variance (ANOVA; using software GraphPad PRISM 9.2). This test calculates differences between operated sides of different experimental groups. To evaluate the differences between (i) operated and unoperated contralateral sides within the same group, and (ii) the operated side of all groups two-way MANOVA was used. A value of *P* < 0.05 was considered to indicate statistical differences for all analyses.

## Results

### Labeling of Facial Motoneurons

The highly constant arrangement of the sensory vibrissae rows (Dörfl, 1982, 1985) provides exact landmarks for reproducible tracer injections into the mimic musculature of the whisker pad (Angelov et al., 1993, 1996, 1997; Streppel et al., 2002; Ozsoy et al., 2014). In intact rats, this injection exclusively labeled motoneurons in the *lateral facial subnucleus*, *the function of which is whisking*, i.e., the rhythmical sweeping movement of the sensory vibrissae.

#### Normal Values

In a pilot experiment we labeled 1,236 ± 72 motoneurons by application of DiI crystals to the proximal stump of the freshly cut buccal branch of the facial nerve (mean ± *SD*; *n* = 6 rats). In several studies, the intramuscular injection of a single tracer into the whisker pad has labeled 1,310 ± 83 motoneurons (weighted mean of means, Table 1). These neurons are localized exclusively in the lateral facial subnucleus (Figure 1A; see for HRP-labeling: Figure 1 in Angelov et al., 1996) and project into the whisker pad through the buccal branch of the facial nerve.

**TABLE 1:** Lateral facial subnucleus.

References	Tracer employed	Mode of tracer application	Number of facial nuclei (and of rats)	Strain of rats	Number of labeled neurons (mean ± *SD*)
Angelov et al. (1993)	HRP	i.m. Injection	6 (of 6)	Wistar	1,254 ± 54
Angelov et al. (1996)	HRP	i.m. Injection	6 (of 6)	Wistar	1,280 ± 113
Streppel et al. (1998)	HRP	i.m. Injection	12 (of 6)	Wistar	1,263 ± 54
Angelov et al. (1999)	DiI	Crystals to stump	4 (of 4)	Wistar	1,724 ± 375
Dohm et al. (2000)	FG	Crystals to stump	6 (of 6)	Wistar	1,324 ± 29
Popratiloff et al. (2001)	DiI	i.m. Injection	6 (of 3)	Wistar	1,317 ± 84
	FB	i.m. Injection	6 (of 3)	Wistar	1,215 ± 55
	FG	i.m. Injection	6 (of 3)	Wistar	1,230 ± 59
Franchi et al. (2006)	HRP	i.m. Injection	9 (of 9)	Wistar	1,140 ± 209
Guntinas-Lichius et al. (2002)	FG	i.m. Injection	6 (of 6)	Lewis	1,472 ± 71
Skouras et al. (2002)	FG	Crystals to stump	10 (of 10)	Wistar	1,746 ± 375
Streppel et al. (2002)	FG	Crystals to stump	9 (of 9)	Wistar	1,422 ± 39
Tomov et al. (2002)	FG	i.m. Injection	12 (of 6)	RCS	1,401 ± 111
This study	DiI	Crystals to stump	6 (of 6)	Wistar	1,236 ± 72
Weighted mean of means		i.m. Injection	60 (of 39)		1,310 ± 83
		Crystals to stump	35 (of 35)		1,500 ± 208

*Normal number of motoneurons innervating the whisker pad through the buccal branch and responsible for movement of sensory vibrissae.*

Using preoperative and post-operative double labeling on the unoperated control side, we counted less than 1,300 neurons in all three groups of this experiment (Table 2). In the same rats that had been injected with 100 μl 1% FG, 76 days prior to perfusion and with 100 μl 1% FB at exactly the same site 66 days later, i.e., 10 days prior to perfusion (cf. Table 2), we counted only 667 ± 148 FG-prelabeled neurons (mean ± SD; *n* = 17), but 1,147 ± 174 FB-post-labeled neurons. In all groups, we found considerably less FG-prelabeled than FB-post-labeled neurons (Table 2 and Figures 1B–D). These data sets are easily explained by the neuron cell bodies containing both FG and FB. The bright blue fluorescence of the latter outshines and masks the red fluorescence of the former, causing a false negative and had decreased the number of FG-labeled cells (Popratiloff et al., 2001). In light of this unavoidable methodological problem, the number of double-labeled neurons—that theoretically should reach 100% on the unoperated side—ranged from 93 to 97% (Table 2) of the number of prelabeled neurons.

**TABLE 2:** Systemic drug treatment by nimodipine or FK506 (Tacrolimus) after implantation of an artificial nerve conduit for nerve repair.

Group of animals	Experimental side (implantation of artificial nerve conduit)			Side difference (FB_op_/FB_cont_)	Unoperated contralateral side		
	FG_op_	FB_op_	FG_op_+FB_op_ (% of FG_op_)		FG_cont_	FB_cont_	FG_cont_+FB_cont_ (% of FG_cont_)
PT (*n* = 6)	782 ± 132	1,308 ± 293	296 ± 62 (38%)	107% ± 13%	742 ± 105	1,213 ± 118	700 ± 98 (94%)
NT (*n* = 6)	691 ± 136	1,259 ± 183	269 ± 56 (40%)	107% ± 19%	709 ± 144	1,192 ± 193	688 ± 143 (97%)
FKT (*n* = 5)	679 ± 73	1,223 ± 122	261 ± 44 (39%)	122% ± 14%	526 ± 87	1,013 ± 124	490 ± 76 (93%)

*Number of single and double-labeled facial motoneurons after injection of FG as prelabeling and FB as post-labeling into the whisker pad muscle with either placebo, nimodipine or FK506 treatment, PT, Placebo Treatment, NT, Nimodipine Treatment, FKT, FK506 Treatment. Each value is the mean ± SD.*

#### Artificial Nerve Conduit—Placebo Treatment

As placebo treatment the control animals were fed standard food pellets, i.e., no treatment in addition to the nerve surgery. Injection of FG into the whisker pad 10 days prior to insertion of the facial nerve conduit and injection of FB 56 days after insertion had prelabeled 782 motoneurons confined within the lateral facial subnucleus and post-labeled 1,308 motoneurons (Table 2, PT). These neurons were spread throughout the lateral, dorsal and intermediate facial subnucleus (Figure 1B), but were not found in the medial facial subnucleus, the axons of which run through the posterior auricular nerve branch that had not been transected during implantation of the nerve conduit. Thus, the distribution of post-labeled neurons and the misdirection of reinnervation were the same after conduit implantation, as well as after direct facial nerve suture (Figure 2 in Angelov et al., 1996, 1999). Only 296 motoneurons were double-labeled, i.e., 38% of the original motoneurons of the whisker pad that had been prelabeled before surgery.

#### Artificial Nerve Conduit—Nimodipine Treatment

Immediately after operation, oral treatment was started with 1,000 ppm nimodipine in food pellets for 56 days (see Figure 2) as previously reported (Angelov et al., 1996, 1997; Guntinas-Lichius et al., 1996, 1997; Mattsson et al., 1999, 2001, 2005). We counted 691 FG-prelabeled and 1259 FB-post-labeled motoneurons (Table 2, NT), of which only 269 motoneurons were double-labeled, suggesting that 40% of the regenerated motor axons correctly reinnervated the original target. As in the placebo group PT, all FG-prelabeled neurons were exclusively localized in the lateral facial subnucleus indicating correct myotopic distribution, whereas the FB-post-labeled neurons were scattered throughout the entire facial nucleus (except for the medial facial subnucleus) with complete loss of myotopic organization pointing to aberrant reinnervation (Figure 1C).

#### Artificial Nerve Conduit—FK506

FK506 was administered by subcutaneous injection in the dose of 1.0 mg/kg body weight per 48 h. On the experimental side, 679 FG-prelabeled and 1223 FB-post-labeled motoneurons were counted after systemic treatment with FK506. 261 motoneurons, i.e., 39% of the FG-prelabeled motoneurons were double-labeled also with FB (Table 2, FKT). The myotopic distribution of FG-prelabeled neurons in the lateral facial subnucleus and of FB-post-labeled neurons were scattered throughout the facial nucleus (Figure 1D) while their distribution was not distinguishable from that of the rats treated with nimodipine or placebo.

Statistical evaluation of the data summarized in Table 2 (two-way MANOVA) proved significance (*P* < 0.05) of the clear difference in the number of double-labeled neurons on the operated side (261–296, respectively, 38–40%) and the unoperated side (490–700, respectively, 93–97%; Figures 1A–D) in the three groups PT, NT and FKT. There was, however, no difference in the number of pre-, post- or double-labeled neurons between placebo, nimodipine or FK506 treated rats on the operated side (Figures 1B–D). *Neither nimodipine nor FK506 improved the accuracy of reinnervation*.

### Biometric Analysis of Vibrissae Movements (Whisking)

Awake rats display whisking, a rhythmic sweeping movement of active muscular protraction and passive elastic retraction of the whiskers (sensory vibrissae), which can be measured by video-based motion analysis as an indicator for the mimetic function.

#### Normal Intact Values

**FIGURE 3:**
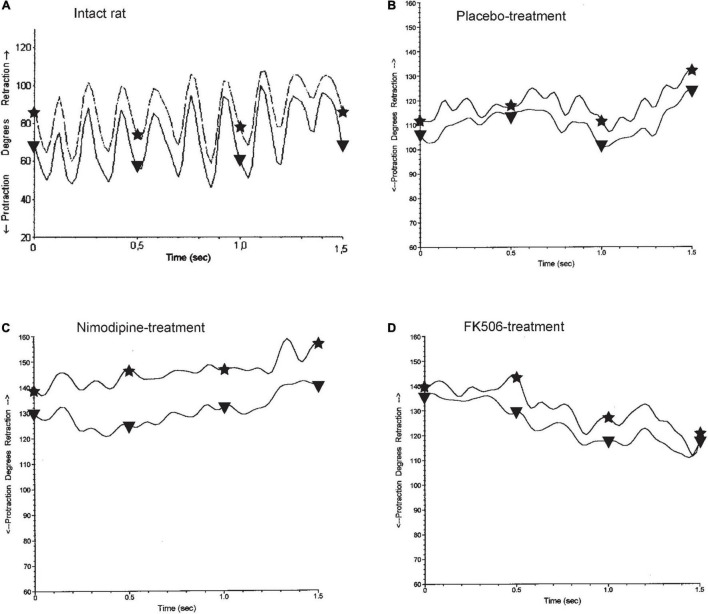
Whisking in normal and experimental rats. **(A)** In the normal intact rats, both vibrissae of the same side show the synchronous sweeping movement of active protraction (*asterisk*) and passive retraction (*triangle*) anhat is characteristic for explorative behavior adopted with permission from Guntinas-Lichius et al. (2002). The graph shows approximately 6 cycles in 1 s, fitting to the measured mean frequency of 6.0 Hz (cf. Table 3), 56 days after implantation of an artificial nerve conduit **(B–D)**. The amplitude and the speed of whisking are notably reduced and the average position of the vibrissae is at a greater angle toward retraction, as active due to mimic musculature protraction instability, which counterbalances passive retraction by elastic connective tissue. Neither treatment of placebo **(B)**, nimodipine **(C)**, nor FK506 **(D)** improved the functional outcome. **(A)** normal intact rat, **(B–D)** operated side 56 days after artificial nerve conduit implantation.

During exploratory behavior intact rats that had not underwent surgery or drug treatment (Table 3), showed 6 cycles of spontaneous protraction and retraction per second (frequency 6.0 ± 0.9 Hz; Figure 3A). At maximal protraction the rostral angle between the longitudinal axis of the body and the test whisker reached a minimum of 74° ± 14°. Higher values of this angle in experimental conditions suggest a reduced ability of the whisker pad muscle to pull the whisker forward. The amplitude of whisking was measured as the angle between the most forward and most backward position of the test whisker during both protraction and retraction. The standard amplitude was 46° ± 13°. The speed (460°/s) and acceleration (16,800°/s^2^) of the angular whisking movement were the maximal measurements that occurred during protraction, i.e., during the phase of active muscle contraction.

**TABLE 3:** Biometrics of restorating whisking behavior 56 days after implantation of the artificial nerve conduit plus placebo, nimodipine or FK506 treatment.

Group of animals	Frequency (Hz)	Angle at maximal protraction (degrees)	Amplitude (degrees)	Maximal angular velocity during protraction (degrees/s)	Maximal angular acceleration during protraction (degrees/s^2^)
Intact rats (*n* = 6)	6.0 ± 0.9	74 ± 14	46 ± 13	461 ± 295	16,796 ± 13,694
Intact side of rats with conduit (*n* = 17)	6.1 ± 1.0 (102%)	91 ± 17 (123%)	51 ± 15 (111%)	1,228 ± 725 (266%)	41,232 ± 35,383 (245%)
PT (*n* = 6)	6.0 ± 0.6 (100%)	115 ± 10 (155%)	15 ± 7 (33%)	269 ± 107 (58%)	7,080 ± 3,492 (42%)
NT (*n* = 6)	5.6 ± 0.9 (93%)	131 ± 11 (177%)	15 ± 5 (33%)	223 ± 85 (48%)	5,856 ± 3,062 (35%)
FKT (*n* = 5)	6.0 ± 0.7 (100%)	113 ± 5 (153%)	22 ± 6 (48%)	251 ± 135 (54%)	6,441 ± 4,726 (38%)

*Each value is the mean ± SD. Data in brackets: (percentage in relation to normal rats).*

**On the unoperated left side of placebo-, nimodipine- and FK506-treated rats**, with an artificial nerve conduit on the right side (Table 3) the whisking frequency was similar to control conditions (6.1 Hz). However, the whisking amplitude in all groups was elevated to 111% of the normal value (51°), the angle at the maximal protraction was increased by 123% (91°), the maximal velocity by 266% (1,200°/s) and the maximal acceleration amounted to 245% (41,000°/s^2^) of control values. These elevations on the intact side most likely reflect compensation of the functional loss on the operated side.

**On the operated right side of all experimental rats** (Table 3), treated with either placebo, nimodipine or FK506 (Figures 3B–D), the whisking frequency ranged from 93 to 100% of the standard value (5.6–6.0 Hz). The whisking amplitude was reduced to 33–48% (15–22°) of the standard 46°. The whisking velocity was decreased to 48–58% (223–269°/s) of the preoperated value 460° and the angular acceleration had dropped to 35–42% (5,856–7,080°/s^2^) of the value of intact rats (16,800°/s^2^), but the angle at maximal protraction had increased to 153–177% (113–131°) indicating a severe impairment of active movement. Analysis of the data by means of ANOVA did not reveal any significant differences between the rats treated with placebo (PT), nimodipine (NT) or FK506 (FKT) after surgery (Figures 3B–D).

*In comparison to placebo, neither nimodipine nor FK506 had beneficial effects on the accuracy of reinnervation and recovery of motor function after facial nerve repair*. These findings support our data on retrograde labeling, where the drug application achieved similar results as observed in the control group (Table 2).

## Discussion

Traumatic injury of the facial nerve leads to paralysis of the mimic musculature. After surgical reconstruction of the continuity of the injured nerve, regeneration occurs and leads to reinnervation. However, this reinnervation—although successful at the cytological level—is misdirected and hence does not yield satisfactory recovery of function: “Despite more than 100 years of intense laboratory and clinical investigations, results of nerve repairs are somewhat discouraging with only 50% of patients regaining useful function” (Lee and Wolfe, 2000). We aimed to improve the accuracy of reinnervation and the quality of functional recovery. To this end, we tested nimodipine and FK506 in rats, as it is already known that these two different drugs accelerate axon elongation in regenerating motoneurons.

Morphologically we measured the accuracy of mimetic muscle reinnervation by sequential double-labeling and counting of the neurons, i.e., by the retrograde fluorescent labeling of motoneurons *first* 10 days before lesion of the facial nerve (prelabeling of the original correct motoneuron pool) and *second* 56 days after facial nerve repair (post-labeling the regenerated motoneuron pool innervating the same muscle). If the post-labeled neurons matched to the prelabeled neurons (100% double labeling), then fully correct reinnervation would have occurred.

Functionally, we analyzed the spontaneous exploratory whisking of sensory vibrissae to assess the recovery of motor function in digital video films with the PEAK Motus 2000 system (Guntinas-Lichius et al., 2002, 2007; Tomov et al., 2002; Franchi et al., 2006; Angelov et al., 2007; Ozsoy et al., 2014; Sofroniew and Svoboda, 2015).

### Sequential Double Labeling and Counting

Retrograde labeling and counting of neurons are well-established methods. Several investigators have employed the sequential application of two tracers to the same nerve branch or target muscle in rats (Brushart, 1990; Molander and Aldskogius, 1992; Madison et al., 1996; Popratiloff et al., 2001; Katada et al., 2006; Ozsoy et al., 2014; Placheta et al., 2015). Selective fluorescence filters to separate FB and FG labeled neurons were used as described (Popratiloff et al., 2001).

In every third 50 μm-section through the brainstem, all fluorescent neuronal cell bodies were counted. Accordingly, we sampled one third of the total volume of the facial nucleus accordingly, using a robust fractionator (Gundersen, 1986; Neiss et al., 1992). The reliability of our morphological methods was demonstrated by the high rate of double labeling on the unoperated contralateral side in our experiments. Guntinas-Lichius and Neiss (1996) reported that counting one third of the rat’s facial nucleus motoneurons through the brainstem, as performed in the present study, the maximum empirical counting error amounted less than 4% aberration of the entire amount of neurons contained in the facial nucleus.

### High Rate of Double Labeling on the Unoperated Contralateral Side

On the unoperated, contralateral side of our experimental rats, 93–97% of the motoneurons were double-labeled in the lateral facial subnucleus after the sequential injection of FG and FB as first and second tracer into the whisker pad. This data coincides well with sequential double labeling on the intact/control side in several studies: 96% (Madison et al., 1996), 77% (Bodine-Fowler et al., 1997), 90–91% (Skouras et al., 2002), and 68% (Katada et al., 2006). Significant lower rates of double labeling, but severe misdirection of reinnervation were observed on the experimental side of all our animals after transection and regeneration of the main facial nerve trunk.

### Pharmacotherapy Did Not Improve Accuracy of Reinnervation, but Modified Hyperinnervation (Polyinnervation)

#### Nimodipine

Administered after facial nerve reconstruction, has some other—presumably beneficial—effects, i.e., neuroprotective effects on motoneurons, astroglia, microglia, and Schwann cells (Guntinas-Lichius et al., 1996, 1997; Angelov et al., 1998; Mattsson et al., 1999; Jia et al., 2015; Sin et al., 2018; Leisz et al., 2019). Nimodipine treatment in rats, following *crush* of the recurrent laryngeal nerve (Hydman et al., 2007) or the facial nerve (Mattsson et al., 2001) has been shown to have positive, clinical observations on the time course, as well as amplitude of the compound muscle action potentials, and visual score of vibrissae movement. Treatment with 1,000 ppm nimodipine in food pellets is the standard post-operation dosage used by previous authors mentioned, as well as in our experiments.

In general, crushed nerves regenerate quite well (Lundborg, 2004). Our data on nimodipine therapy after facial *nerve transection*, do not support the favorable findings of Mattsson et al. (2001) working in a crush model. We measured amplitude of whisking by quantitative motion analysis (data in brackets: amplitude). The amplitude was decreased from 46° as in intact rats, to 15° (-67%) in both groups: facial nerve conduit plus placebo and facial nerve conduit plus nimodipine. This value was also reduced to 22° (-52%) in those rats with facial nerve conduit plus FK506 (Table 3 and Figure 3). A similar finding was reported by Franchi et al. (2006) in newborn rats after facial axotomy and repair, in which the reorganized vibrissal representation was reduced to the medialmost portion of the normal vibrissal representation. Hence, in our transection model the regeneration of motor function presented the same results, as without nimodipine therapy and vibrissae whisking was even significantly worse.

With regard to function, nimodipine was not beneficial for the rats in our experiment. Concerning morphology, nimodipine did not perform better. Following conduit reconstruction of the facial nerve, 38% of the neurons were double-labeled with placebo and 40% with nimodipine (Table 2). In our experiments, nimodipine also did not reduce hyperinnervation. Hyperinnervation was first described in the study of Angelov et al. (1993) on hypoglossal-facial anastomosis describing a higher reinnervation of distinct target muscles after nerve repair and regeneration, e.g., in the whisker pad, than on the contralateral normal side. After direct facial nerve suture, a hyperinnervation of 132.6% in young adults (Angelov et al., 1996) and 160.2% in 2-year-old rats (Streppel et al., 1998) were observed. With nimodipine treatment, the hyperinnervation was reduced to 113% in young adult rats (Angelov et al., 1996, 1997). In the present investigation, however, there was little to no detection of hyperinnervation in the artificial nerve conduit. Hyperinnervation was only 107% in both the placebo and nimodipine groups (Table 2). The data gathered can be interpreted as a lack of excessive sprouting in both groups, rather than a failure in nimodipine’s ability to reduce hyperinnervation.

#### FK506 (Tacrolimus, Prograf^®^)

Is an immunosuppressive drug that also dose-dependently accelerates neuronal regeneration (Gold et al., 1995, 1998, 1999; Doolabh and Mackinnon, 1999; Fansa et al., 1999; Gold, 1999; Wang and Gold, 1999; Jost et al., 2000; Lee et al., 2000; Navarro et al., 2001; Chunasuwankul et al., 2002; Sulaiman et al., 2002; Udina et al., 2003; Konofaos and Terzis, 2013; Shahraki et al., 2015; Labroo et al., 2016; Huang et al., 2017; Jo et al., 2019). FK506 can bind immunophilin neuronal receptors, causing activation of heat-shock proteins and, in turn, increased expression of growth-associated genes, i.e., growth-associated protein (GAP)-43, and activation of the mitogen-activated protein kinase pathways (Gold et al., 1995, 2004; Huang et al., 2017; Jo et al., 2019). Further, FK506 protects against neuronal cell death (Winter et al., 2000; Sosa et al., 2005; Konofaos and Terzis, 2013). The immunosuppression is based on inhibition of the calcium/calmodulin-dependent phosphoserine/phosphothreonine protein phosphatase calcineurin by the complex of FK506 and the 12-kDa FK506-binding protein (FKBP-12). As the immunosuppressive Cyclosporin A likewise inhibits calcineurin, but does not improve neural regeneration, FK506’s nerve regenerating property must involve a distinct, calcineurin-independent mechanism (Wang et al., 1997; Gold, 1999). Hence, non-immunosuppressive ligands to FK506-binding proteins were sought to elicit the neuroprotective/neurotrophic effect of FK506. One such compound, GPI-1046 has proved useless in a neuroprotection assay (Winter et al., 2000).

In our experiments, FK506 had no positive functional effects but showed side effects. Despite the subimmunosuppressive dose of 1 mg FK506/kg bw/48 h (Yang et al., 2003), one rat developed an abscess at the injection site in the neck region, then a peritoneal abscess/peritonitis and was lost during treatment. 56 days after implantation of an artificial nerve conduit, whisking was as reduced under FK506 treatment, as well as the placebo and nimodipine therapy (Table 3 and Figure 3). Regarding accuracy of reinnervation, 39% of the neurons that had originally innervated the whisker pad, were double-labeled following FK506 treatment, while only 40% of neurons were double-labeled after nimodipine and 38% following placebo treatment (Table 2). FK506-treated rats only differed from those of the placebo and nimodipine group in that they showed a much higher hyperinnervation (122 vs. 107%, Table 2).

The increase of hyperinnervation, i.e., an increase of axonal branching that physiologically occurs during nerve regeneration (Shawe, 1955; Brushart et al., 1998; Al-Majed et al., 2000; Jiang et al., 2007) is probably due to a neurotrophic effect. FK506 has been reported to raise the GAP-43 expression in axon sprouts of dorsal root ganglion cells (Gold et al., 1998), and to stimulate the proliferation of Schwann cells *in vitro* (Fansa et al., 1999). In rodents, FK506 was shown to increase the number of axons in the regenerating nerve (Chunasuwankul et al., 2002; Sobol et al., 2003; Yang et al., 2003; Brenner et al., 2004; Huang et al., 2017) while others did not observe such results (Fansa et al., 1999; Navarro et al., 2001; Udina et al., 2003). Neuroregenerative properties of FK506 are in line with hyperinnervation, however, it certainly does not improve the recovery of motor function. Congruent with our unsuccessful result, it has been reported that ultimately no functional recovery was achieved in rats after experimental nerve injury and treatment with FK506 (Doolabh and Mackinnon, 1999; Fansa et al., 1999). Except for the study of Brenner et al. (2004), walking track analysis after sciatic or tibial nerve transection and repair did not reveal noticeable differences between FK506- and placebo-treated rodents at the end of the observation periods (Jost et al., 2000; Navarro et al., 2001; Sobol et al., 2003). Brenner et al. (2008) have reported that benefits of FK506 in sciatic nerve regeneration as observed in comparison to placebo at 40 days post-operation had disappeared at 70 days post-operation. We have used 56 days regeneration time as endpoint of this study, as previous studies up to 112 or 224 days post-suture (Angelov et al., 1993; Streppel et al., 1998) have shown that at 56 days after facial nerve repair the regeneration of motoneurons is almost complete in rats, and obviously only then final function can be judged. In summary, our data suggest that acceleration of regeneration does not necessarily entail improvement of functional recovery.

## Conclusion

Previous studies have shown that nimodipine appears to have the potential to accelerate axon regeneration and the immunosuppressant FK506 potentially improves hyperinnervation. However, in this study; neither nimodipine- nor FK506 treatment showed significant effects on the accuracy of reinnervation. Misdirection of reinnervation remains a major problem, severely compromising ultimate functional recovery. The facial nerve model with conduit reconstruction in rodents, which may or may not be generalizable to peripheral nerve lesions i.e., sciatic nerve and to large animals/humans.

## Data Availability Statement

The raw data supporting the conclusions of this article will be made available by the authors, without undue reservation.

## Ethics Statement

The experimental animal study was reviewed and approved by the Bezirksregierung Köln (Az. 23.203.2-K 35, 18/00) based on the guidelines of the European Union Council (86/609/EU) and according to § 8 Tierschutzgesetz (German Federal Law for the Protection of Animals).

## Author Contributions

MB designed, perfused the animals, counted motoneurons, and wrote the manuscript with contributions from WN and MS operated the animals, and analyzed statistical data. OG-L operated the animals and interpreted data. JV interpreted and analyzed statistical data. NF-S interpreted data. WN designed, analyzed data, as well as edited the manuscript, and supervised this research in his laboratory. All authors contributed to the article and approved the submitted version.

## Conflict of Interest

The authors declare that the research was conducted in the absence of any commercial or financial relationships that could be construed as a potential conflict of interest.

## Publisher’s Note

All claims expressed in this article are solely those of the authors and do not necessarily represent those of their affiliated organizations, or those of the publisher, the editors and the reviewers. Any product that may be evaluated in this article, or claim that may be made by its manufacturer, is not guaranteed or endorsed by the publisher.
